# Metacommunity dynamics of bacteria in an arctic lake: the impact of species sorting and mass effects on bacterial production and biogeography

**DOI:** 10.3389/fmicb.2014.00082

**Published:** 2014-03-04

**Authors:** Heather E. Adams, Byron C. Crump, George W. Kling

**Affiliations:** ^1^Department of Ecology and Evolutionary Biology, University of MichiganAnn Arbor, MI, USA; ^2^College of Earth, Ocean and Atmospheric Science, Oregon State UniversityCorvallis, OR, USA

**Keywords:** aquatic microbiology, arctic, bacterial production, species sorting, mass effects, metacommunity theory, transplant experiments

## Abstract

To understand mechanisms linking ecosystem processes and microbial diversity in freshwater ecosystems, bacterial productivity and the metacommunity dynamics of species sorting and mass effects were investigated in an 18 ha headwater lake in northern Alaska. On most sampling dates, the phylogenetic composition of bacterial communities in inflowing streams (inlets) was strikingly different than that in the lake and the outflowing stream (outlet) (16S DGGE fingerprinting), demonstrating the shift in composition that occurs as these communities transit the lake. Outlet and downstream communities were also more productive than inlet and upstream communities (^14^C-leucine incorporation). Inlet bacteria transplanted to the outlet stream in dialysis bags were equally or less productive than control bacteria, suggesting that the inlet bacteria are capable of growing under lake conditions, but do not remain abundant because of species sorting in the lake. Outlet bacteria (representative of epilimnetic bacteria) transplanted to the inlet stream were less productive than control bacteria, suggesting that lake bacteria are not as well adapted to growing under inlet conditions. Based on water density, inlet stream water and bacteria generally entered the lake at the base of the epilimnion. However, during low to medium flow in the inlet stream the residence time of the epilimnion was too long relative to bacterial doubling times for these allochthonous bacteria to have a mass effect on the composition of outlet bacteria. The highest community similarity between inlet and outlet bacteria was detected after a large rain event in 2003, with over 61% similarity (average non-storm similarities were 39 ± 8%). While mass effects may be important during large storm events, species sorting appears to be the predominant mechanism structuring bacterial communities within the lake, leading to the assembly of a lake community that has lost some ability to function in stream habitats.

## Introduction

Dispersal and competition are two fundamental mechanisms that influence the presence and dominance of populations within biological communities including plants, animals, and microorganisms. In aquatic systems, a primary dispersal mechanism for bacterial communities is water flow from terrestrial soils into lakes and streams, resulting in a mixture of communities and resources that may favor certain populations or alter overall community growth (e.g., Crump et al., 2012). Dormant and slow-growing bacterial cells can become active when their preferred carbon and nutrient resources appear (Judd et al., 2006; Jones and Lennon, 2010; Lennon and Jones, 2011; Gibbons et al., 2013), and previously active cells may be at a competitive disadvantage given the new mix of substrates input from upstream. Over relatively short time scales (minutes to months), dispersal via advection and selective competition among organisms (i.e., species sorting) generate microbial biogeographic patterns across aquatic ecosystems and landscapes (Hanson et al., 2012; Logue et al., 2012), but little is known about the relative importance of the processes generating these biogeographic patterns (e.g., Logue and Lindstrom, 2010) or about how these patterns in diversity affect bacterial function in ecosystems.

Metacommunity theory incorporates the mechanisms of dispersal and competition into four main perspectives that act alone or interact within a habitat: species sorting, mass effects, patch dynamics, and neutral processes (Leibold et al., 2004). Species sorting emphasizes spatial niche separation where relatively low levels of dispersal allow communities to respond to local conditions (Leibold and Wilbur, 1992). In contrast, mass effects allow inferior competitors to persist in the community due to high levels of dispersal from other habitats (Urban, 2004). Patch dynamics defines habitat patches as identical with local species diversity determined by dispersal or species interactions, and requires trade-offs in species traits for regional co-existence to occur (Mouquet et al., 2005). Neutral theory assumes functional equivalency among species such that patterns in diversity are non-deterministic and driven by immigration and chance (Sloan et al., 2006; Lindström and Langenheder, 2012). Mass effects and species sorting are thought to be most applicable to aquatic bacterial communities for several reasons: bacteria are easily dispersed by water flow and have potentially high rates of immigration and emigration (Lindström and Bergström, 2004), environment patches are heterogeneous, especially at the microbial scale (Scheffer et al., 2003), bacterial community composition can shift on very short time scales (e.g., days to weeks; Judd et al., 2006; Van der Gucht et al., 2007; Hornak and Corno, 2012), and bacterial populations differ in growth rates and metabolic capabilities (Amon and Benner, 1996; Lapara et al., 2002; Bertoni et al., 2008; Adams et al., 2010). The relative importance of these mechanisms in controlling bacterial community assembly depends on traits of bacterial populations such as resource specialization and dispersal ability (Lindström and Langenheder, 2012). The persistence of bacterial populations that immigrate via water to a new habitat will depend on the number of cells dispersed to that habitat, and their ability to outcompete pre-existing populations through growth and avoidance of grazing or viral lysis.

Time scales of bacterial growth and dispersal also influence whether mass effects or species sorting are most important in structuring a bacterial community (Logue, 2010; Lindström and Langenheder, 2012). In lake catchments, the main mode of bacterial dispersal is likely unidirectional as water flows downhill from upstream soils, groundwater, hyporheic zones, streams, and lakes, although atmospheric deposition can also disperse bacteria (Jones and McMahon, 2009). Bacterial cells entrained in flowing water rely on the water pathways and mixing to reach suitable resources within new habitats. In order for bacteria to establish in a new habitat, they must have a sufficient growth rate to compete with other populations for resources, and population growth must exceed the rate of emigration due to water flow out of a habitat (i.e., mass effect). The physiological response of bacteria to new environmental conditions can be quite fast; e.g., productivity can respond to changes in temperature within 1–2 h (Kirchman et al., 2005; Bertoni et al., 2008; Adams et al., 2010). However, community shifts due to species sorting may take longer than the time that bacterial populations can remain in a habitat, particularly during a storm event with high water flow.

The mechanisms of community assembly at work in these habitats have consequences for processing of DOM and other resources across the landscape. Bacterial response to temperature and substrate- or nutrient-limiting conditions may be specific to the physiological capabilities of the community assemblage present at a given time. For example, soil bacteria transported into aquatic systems along with terrestrial dissolved organic matter (DOM) mix with stream communities that likely contain bacteria accustomed to processing terrestrial DOM. But when this community enters a lake, it encounters lake populations that may be limited in their ability to process terrestrial DOM. For example, Judd et al. (2006) showed experimentally that shifts in community composition along a landscape gradient (upland terrestrial to lowland aquatic ecosystems) occur in response to changes in the available dissolved organic substrates, and in turn the community productivity changes; Crump et al. (2007, 2012) observed these shifts in natural communities along the same landscape gradients. In addition, the community composition originally present may constrain the microbial physiological adjustment to a change in environment or resources (e.g., Comte and Del Giorgio, 2009), but dispersal may alter this original community composition independent of any metabolic response (Crump et al., 2007). Thus, the metacommunity processes have a direct effect on ecosystem function by replacing species and altering the resultant community's ability to process available nutrients and carbon. Here we investigate the role of mass effects and species sorting in structuring bacterial communities by examining the natural variability of habitat conditions and bacterial communities upstream and downstream of a small arctic headwater lake. We also measured the impact of dispersal on both community structure and growth rates of bacterial communities in different habitats. We address three main questions: (1) How do lakes alter habitat characteristics and bacterial community activity and diversity along landscape flowpaths? This is addressed by comparing habitats and bacterial communities upstream and downstream of a headwater lake. (2) Do stream communities remain active in a lake habitat when competition with lake communities is removed? (3) Do lake communities retain the ability to process upstream DOM, or do community shifts in the lake result in the loss of most bacterial populations with this ability? These latter two questions are addressed using transplantation of natural communities in dialysis bags between habitats.

## Materials and methods

### Study site

**Figure 1:**
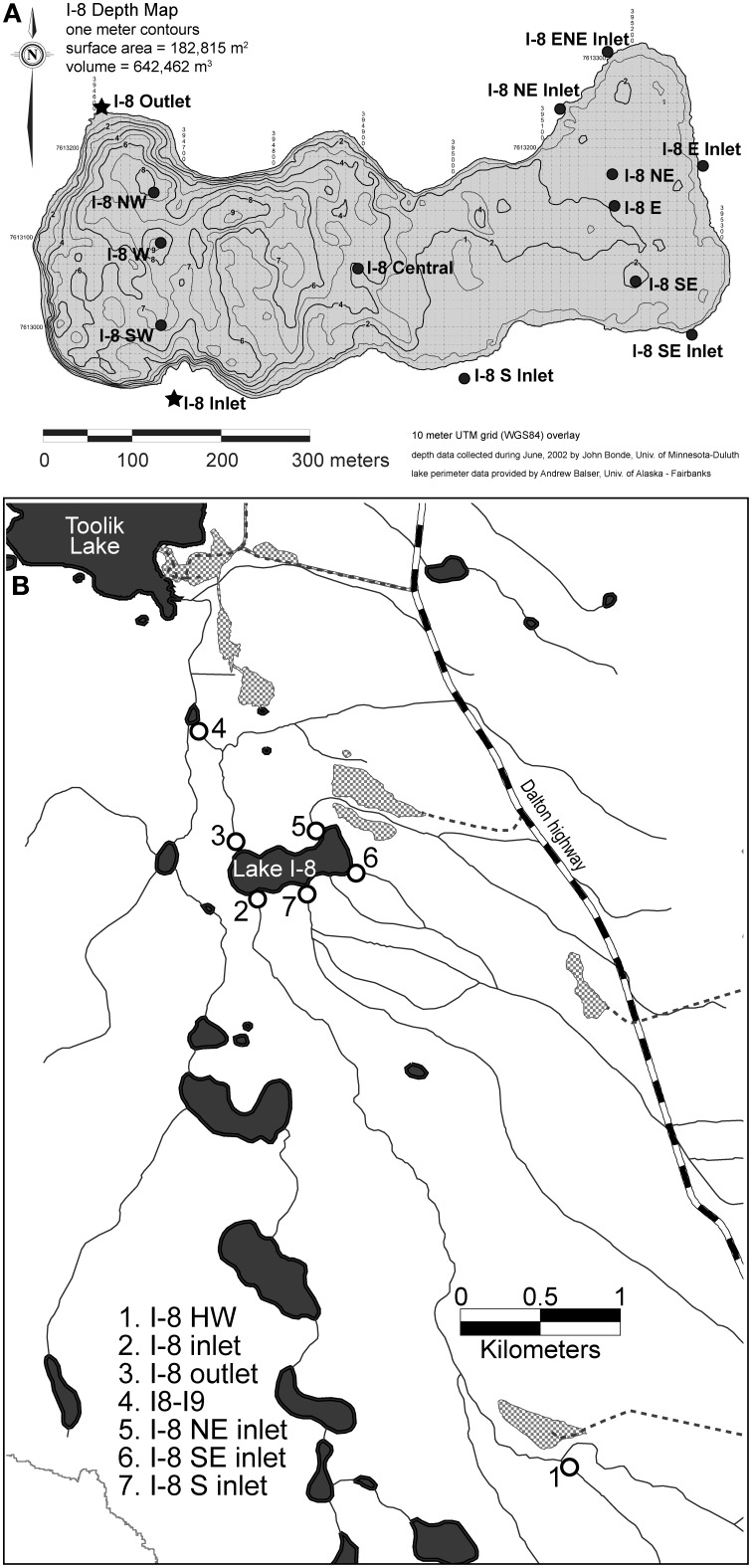
**(A)** Lake spatial sampling sites at Lake I-8, Northern Alaska. I-8 outlet is located at 394605.858 Easting 7613243.017 Northing on the UTM Zone 6 North, WGS84 coordinate system (149.59°W, 68.61°N). **(B)** Sampling locations in the I-series catchment to Toolik Lake, Alaska.

Sites are located on the north slope of the Brooks Range, Alaska, at the Arctic Long Term Ecological Research site. Samples were collected from upstream of, downstream of, and within Lake I-8, which is located two kilometers upstream of Toolik Lake. Lake I-8 is 18.2 ha in area with a volume of 642,500 m^3^ and drains a catchment of 2910 ha. It is oligotrophic, with mean epilimnetic primary productivity of 17.4 μ g C/L/d (range = 2.1–38.8) and mean chlorophyll *a* of 0.92 μ g/L during the ice-free season (Kling et al., 2000). Lake I-8 has a main inflowing (inlet) stream, sampled ~5 km upstream of the lake at the site I-8 HW, as well as where the stream flows into the lake at site I-8 inlet. There are three smaller inlet streams, (I-8 NE inlet, I-8 SE inlet, and I-8 S inlet) that also flow into the lake, and a single outflowing stream (I-8 outlet). Site I8-I9 is one km downstream of the lake outlet (Figure 1). Water temperatures for summers 2003–2007 (June–August) ranged from 3.3 to 18°C (mean = 10.5°C) at I-8 inlet, and ranged from 5.8 to 18.4°C (mean = 12.9°C) at I-8 outlet. There are usually 2–3 storm events during the summer season, post snow-melt.

### Field measurements

Bacterial production, bacterial community composition, temperature, and DOM concentration were measured weekly at Lake I-8 inlet, I-8 outlet, and at site I8-I9 in the summers of 2003–2007 (Figure 1). The smaller inlets to the lake were also sampled weekly during the summer of 2007. I-8 HW stream and the lake itself were sampled three times every summer, with weekly sampling of the lake occurring during the summer of 2003 and a more intense spatial sampling at eight sites across the lake on 4 July 2007 (Figure 1). Stream water samples were collected from mid-stream while avoiding disturbance of streambed surfaces. Lake samples were collected using a Van Dorn bottle sampler, typically at the depths of 1 m (epilimnion) and 6 m (hypolimnion). Temperature was measured with a digital thermometer, conductivity with a model 122 Orion meter, and pH with a model 210A Orion meter. Stream discharge and temperature were monitored at the sites using dataloggers (Onset StowAways and Hobos) and a Marsh-McBirney Flowmate discharge meter. Water residence time (WRT) of the lake was calculated by dividing the lake volume by the mean outlet stream discharge. Epilimnetic WRT was calculated using the lake volume from surface to 3 m, which is the mean thermocline depth during the summer season. Time series temperature measurements in 2003 were obtained from the I-8 W station with seven Brancker TR 1050 self-contained temperature loggers (thermistors) at depths 0.05, 0.53, 1.03, 3.03, 4.03, 5.03, and 6.03 m (installed by MacIntyre). Isotherm depths were determined by linear interpolation of readings taken every 15 min. Thermistors had an accuracy of 0.002°C and a time constant of <3 s (MacIntyre, pers. comm.).

Bacterial production (BP) was measured using ^14^C labeled-leucine uptake following Kirchman (1992), and assuming an intracellular isotopic dilution of 1. Each measure was calculated from incubation of three unfiltered 10 mL subsamples and one 10 mL trichloroacetic acid (TCA) killed control for ~3 h before ending by adding TCA to a final concentration of 5%. Samples were filtered onto 0.2 μm nitro-cellulose filters and extracted using 5 mL of ice-cold 5% TCA. Filters were then dissolved in scintillation vials using ethylene glycol monoethyl ether, flooded with Scintisafe scintillation cocktail and counted on a liquid scintillation counter (Packard Tri-Carb 2100TR).

DOM samples were filtered in the field through ashed Whatman GF/F filters and stored at 4°C until analysis. Protein concentrations were determined within 48 h using the Bradford reagent assay (modified from Bradford, 1976) and phenolic concentrations were determined within 48 h using the Folin-Ciocalteu assay (Waterman and Mole, 1994). DOC and chlorophyll *a* concentrations were determined as in Kling et al. (2000).

Samples for cell counts were preserved with 2.5% final concentration of gluteraldehyde and stored at 4°C until analysis. Samples from 2005 were counted on a FACSCalibur (BD Biosciences) flow cytometer following del Delgiorgio et al. (1996). Sub-samples were stained with SYBR green nucleic acid stain in the dark for a minimum of 15 min (Marie et al., 1997; Lebaron et al., 1998). The concentration of the standard 1 μm bead solution and multiple confirmatory cell count samples were measured by epifluorescence microscopy. Samples from 2006 to 2007 were counted on a LSR II flow cytometer (BD Biosciences) as described by Ewart et al. (2008) with data acquired in log mode for at least 60 s and until 20,000 events were recorded, with the minimum green fluorescence (channel 200) set as the threshold. Cell doubling times were calculated using BP, average cell counts from environmental samples, and a conversion factor of 20 fg C/cell (Lee and Fuhrman, 1987).

Bacterial community composition was measured with denaturing gradient gel electrophoresis (DGGE) of PCR-amplified 16S rRNA genes applied to DNA samples. DNA was collected by filtering ~500 mL of sample through a Sterivex 0.2 μm filter, stored at −80°C, and processed as described fully in Crump et al. (2003) and Adams et al. (2010). Imaging of DGGE gels was performed with Quantity One software on a Chemi-Doc gel documentation system (Bio-Rad) and gel bands were identified using GelCompar software to create a presence-absence matrix as described by Crump and Hobbie (2005). Each band represents an operational taxonomic unit (OTU) of bacteria. Dice transformation (SPSS 14.0 through 17.0) was used to condense presence-absence of OTUs into percent community similarities between samples. PROXCAL (SPSS Categories, versions 14.0 through 17.0) was used to create non-metric multi-dimensional scaling (NMDS) graphs of sample similarities. Two-tailed paired *t*-tests (Excel 2003) were used to compare the number of populations (bands) between sites.

### Transplant experiments

Transplantation of bacterial communities was performed to test activity in different habitats. Dialysis bags (Sigma dialysis tubing cellulose membrane, 76 mm flat width, typical molecular weight cut-off ~14,000 Da) were used to isolate bacterial communities from inputs of new bacteria (Gasol et al., 2002). Substrates smaller than 12,000 Da diffuse across the tubing membrane in less than 18 h, as confirmed with a diffusion test of ^14^C-labeled leucine (Supplemental Figure 1), allowing exposure of the contained bacteria to ambient temperature and nutrient conditions. Bags were washed and soaked in DI water to remove excess glycerin for a minimum of 12 h before use, no sections of tubing were used more than once, and nitrile gloves were worn when handling the bags. At each site, three (two in 2005) replicate samples of whole water were collected in acid-washed 1L opaque Nalgene bottles and either transported to another site within 15 min or immediately transferred to 45.7 cm sections of tubing and closed (final volume 640 mL). Grazers were not removed for these experiments due to co-occurrence with particle-attached bacteria. Filled bags were then secured to dowels with plastic ties within open-topped plastic covered metal cages of dimensions 70 × 55 × 55 cm and secured in streams using rebar. Each of three replicate bags for each treatment was allowed to incubate *in situ* for 2–4 days. Upon collection, the contents of each bag were transferred to an acid-washed 1 L opaque Nalgene bottle from which 40 mL was used to measure bacterial production, 180 mL was filtered to measure chl *a*, proteins, phenolics, and DOC, 10–15 mL was preserved for cell counts, and the remainder was filtered to collect DNA.

Several transplant experiments were conducted at I-8 inlet and outlet. The first set of experiments presented here was performed on 18–21 July 2006 and consisted of incubating the I-8 inlet community at both the inlet and outlet sites to test if the community flowing into the lake would be active downstream. The second set of experiments conducted on 5–9 July 2005 and 2–4 July 2007 included the incubation of the outlet community at inlet and outlet sites to test if the downstream community retained the ability to process DOM from upstream. The third set of experiments conducted on 26–28 July 2005 and 1–3 August 2006 consisted of transplantation of both inlet and outlet communities between sites in addition to controls incubated at their original habitat.

## Results

### Differences in activity and community composition between sites

**Figure 2:**
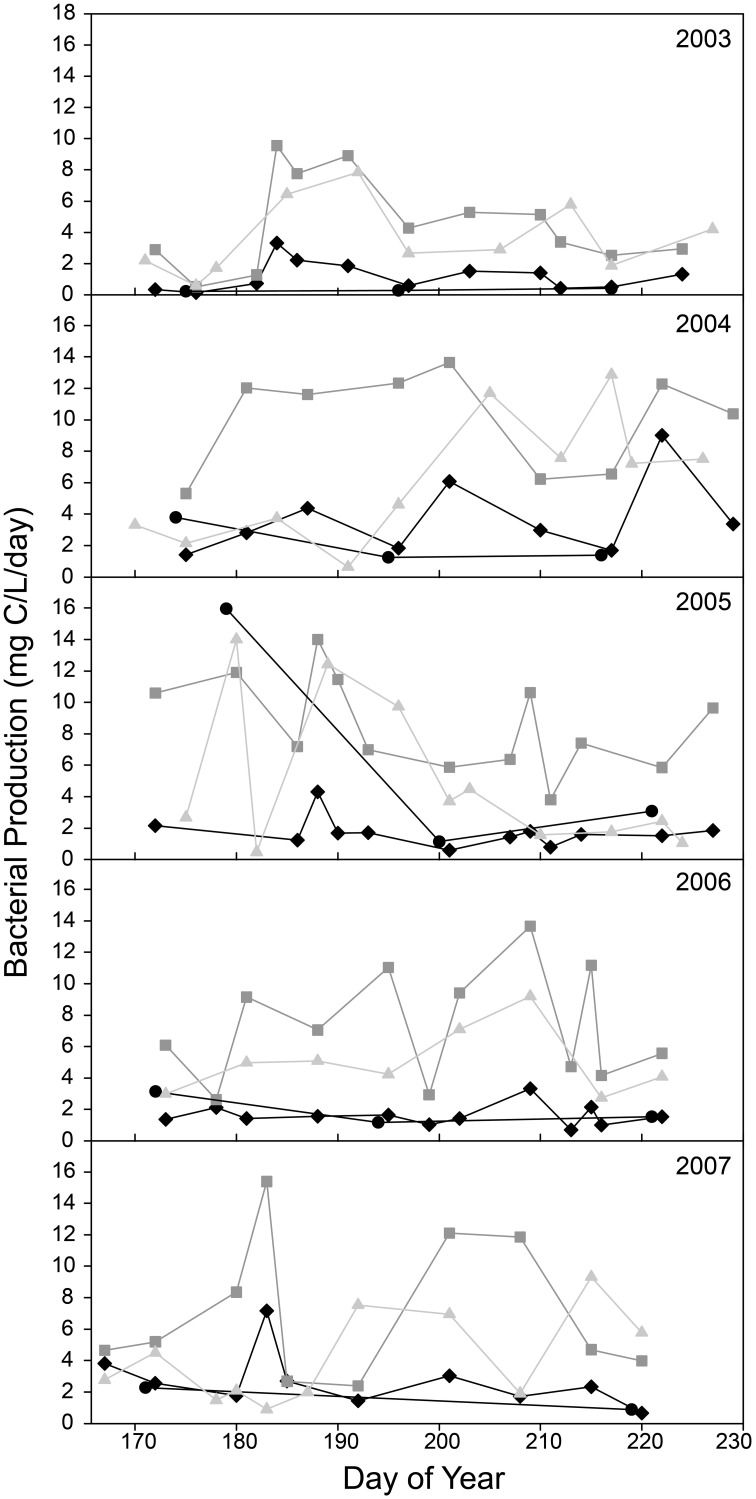
**Bacterial Production at sites upstream and downstream of Lake I-8 for sites I-8 headwaters (black circles), I-8 inlet (black diamonds), I-8 outlet (gray squares), and I-8 to I-9 (gray triangles)**.

During the ice-free summer season, bacterial productivity (BP) was usually greater downstream of Lake I-8 than upstream (Figure 2). At upstream sites from 2003 to 2007, BP averaged 4.3 μ g C/L/d (*SD* = 7.6, *n* = 15) at I-8 HW and 2.1 μ g C/L/d (*SD* = 1.6, *n* = 56) at I-8 inlet. At downstream sites, BP averaged 7.5 μ g C/L/d (*SD* = 3.8, *n* = 57) at I-8 outlet and 4.8 μ g C/L/d (*SD* = 3.4, *n* = 50) ~1 km downstream at site I8-I9.

**Figure 3:**
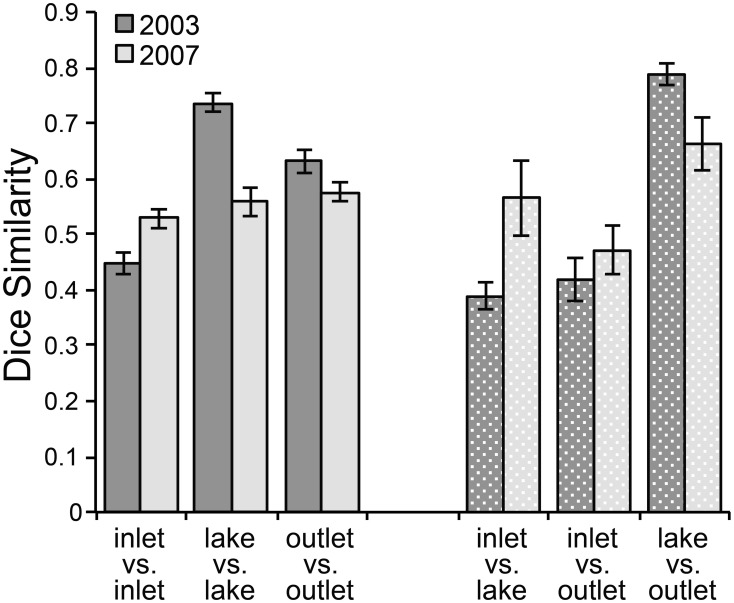
**Dice similarity of DGGE banding patterns at sites at Lake I-8 in summer 2003–2007**. Error bars are standard error of the mean. Solid columns show average pair-wise similarity among communities at each site (inlet, lake, outlet) across sampling dates. Stippled columns show average pair-wise similarities between communities at different sites sampled on the same day. “Inlet” designates the I-8 inlet stream, “outlet” designates the outlet stream, and “lake” designates samples collected from the lake water column at the I-8 West station.

Community composition was also consistently different between sites upstream and downstream of the lake. In 2003, the bacterial community composition at I-8 inlet was variable over time with a low average similarity of 45% between inlet samples (inlet vs. inlet in Figure 3, Supplemental Table 1). Mid-lake and outlet communities were generally more stable during the summer and had average similarities of 74 and 63%, respectively. On each sampling date, the lake and outlet communities (“lake vs. out” in Figure 3) had a very high mean similarity of 79%; this was expected because the outlet is essentially an integrated sample of the lake epilimnion. The inlet communities were less similar to the other two sites (average similarity of ~40%). In 2007, community similarity between sites had a similar pattern but absolute differences were muted. The number of OTUs also differed between sites, with 20 ± 2 bands on average at the inlet and 26 ± 3 at the outlet in 2003 (*p*-value = 0.003). The inlet also had significantly fewer OTUs than the outlet in 2007 with 12 ± 3 bands found at the inlet and 18 ± 4 at the outlet (*p*-value = 0.01).

### Transplant experiments

**Figure 4:**
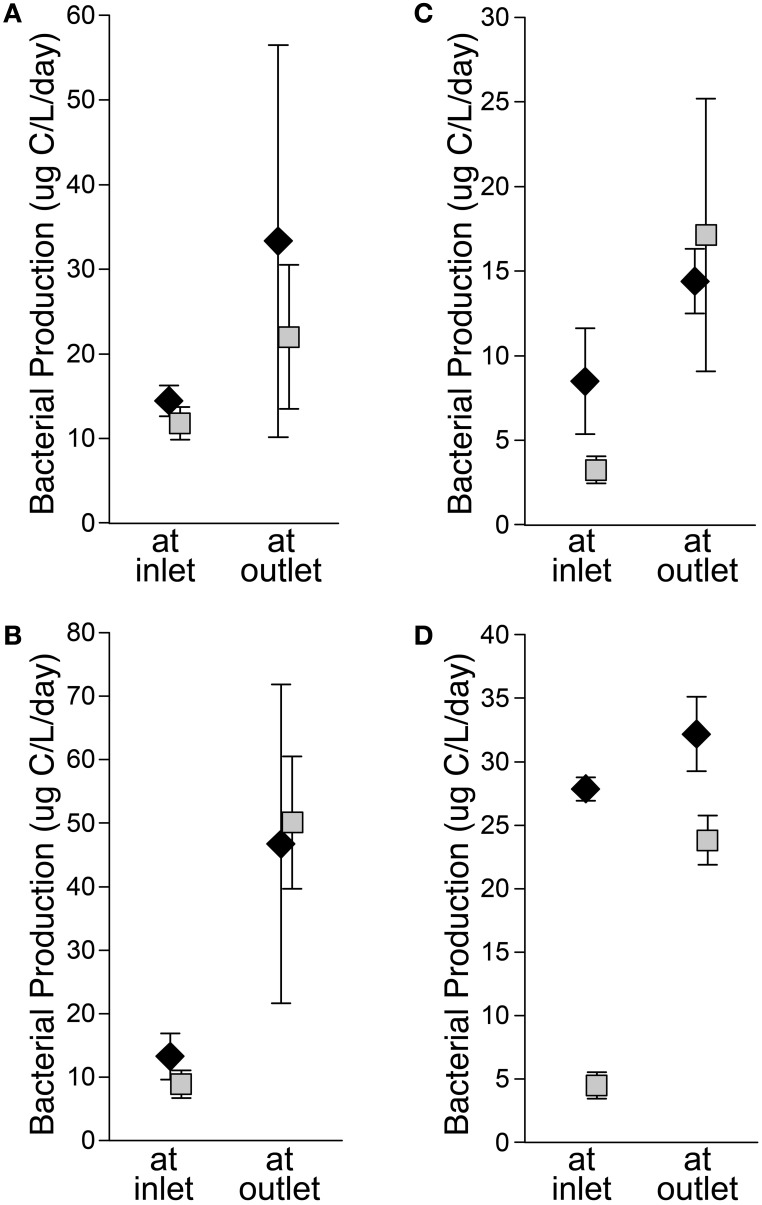
**Left: Early summer transplants at I-8 inlet and outlet from 5 to 9 July 2005 (A) and 2–4 July 2007 (B)**. Right: Late summer transplants at I-8 inlet and outlet from 26 to 28 July 2005 **(C)** and 1–3 Aug. 2006 **(D)**. For all graphs, diamonds are inlet communities and squares are outlet communities. Error bars are standard error of the mean.

Bacteria transplanted between sites showed patterns in activity similar to those observed *in situ*. To test if bacteria flowing into the lake have the potential to be active in lake habitats, bacteria from the inlet were held in place or moved to the outlet, which represented lake conditions (Kling et al., 2000). Bacteria from the inlet (diamonds on Figure 4) incubated at the outlet always had greater bacterial production than control incubations at the inlet, although the difference was not always statistically significant. Bacteria were also moved from the outlet to the inlet to test if the communities that developed across the lake could process stream inlet DOM. Bacteria from the outlet (squares on Figure 4) incubated at the inlet always had depressed activity relative to control incubations at the outlet. In the early season transplants, rates of bacterial production were similar for inlet and outlet communities regardless of incubation location, but in the late season the transplants of inlet communities often had higher bacterial production rates than outlet communities suggesting community-specific responses to treatments. For all transplant experiments, the outlet habitat had more chl *a* and generally more protein and DOC than the inlet habitat (Supplemental Table 2). Temperatures tended to be warmer at the outlet, but transplants were conducted under a range of conditions including nearly equal temperatures and warmer at the inlet. The effect of temperature on bacterial productivity and community composition at these sites was previously found to be complicated due to multiple temperature optima within the communities (Adams, 2010; Adams et al., 2010).

### Lake inputs

The main I-8 inlet is the largest contributor of water to the lake, and in 2007 it had the greatest contribution to inflow when total inflow was highest (Table 1). The other inlets to the lake were either ephemeral (I-8 SE inlet and I-8 E inlet) or had much lower flow compared to the main inlet. I-8 NE inlet accounted for over 36% of water inputs on 4 July 2007, possibly due to rainstorms only in that part of the catchment, but its flow was only 10.2 L/s, which would have been 2% of the total inflow a month later on 3 August 2007 (Table 2). During wetter years, it is anticipated that I-8 inlet accounts for the majority of the water inputs due to its large catchment size of 1281 ha, which is 44% of the total catchment for Lake I-8.

**Table 1:** **Water contribution to Lake I-8 for summer 2007**.

**Date**	**% contribution to lake inflow**				**Inflow (L/s)**	**Outflow (L/s)**
	**I-8 NE inlet**	**I-8 S inlet**	**I-8 SE inlet**	**I-8 inlet**		
16 June 2007					10.2	32.7
18 June 2007	6.4	5.1	1.0	87.4	24.4	34.4
21 June 2007					8.5	32.1
29 June 2007					42.9	41.9
4 July 2007	36.1	6.4	n/a	57.5	28.3	27.1
11 July 2007					11.2	17.9
16 July 2007	14.0	8.7	0.8	76.4	320	47.6
20 July 2007	10.0	8.2	1.2	80.6	134	162
28 July 2007	8.3	8.9	0.9	81.9	48.3	50.7
3 Aug. 2007	5.2	5.8	0.4	88.7	503	619

*Outflow is stream discharge measured at the I-8 outlet. Inflow is the sum of discharge rates for all lake inlets; inflows for dates 16, 21, and 29 June, and 11 July are based only on discharge measured at I-8 inlet*.

**Table 2:** **Water residence time (WRT) for Lake I-8 and doubling times (DT) for I-8 inlet and outlet bacteria, based on data from 2003 to 2007**.

**Year**	**Lake WRT (days)**			**Epilimnetic WRT (days)**		
	**Baseflow**	**Mean**	**Storms**	**Baseflow**	**Mean**	**Storms**
2003	120	3.9	1.0	89	2.9	0.8
2004	122	5.8	1.3	90	4.3	1.0
2005	811	20	1.5	600	15	1.1
2006	39	9.4	2.1	29	6.9	1.5
2007	328	23	2.7	243	17	2.0
Average	284	12.5	1.7	210	9.3	1.3
**Site**	**Longest DT (days)**			**Mean DT (days)**	**Shortest DT (days)**	
I-8 inlet	30			1.9	0.4	
I-8 outlet	12			0.9	0.4	

**Figure 5:**
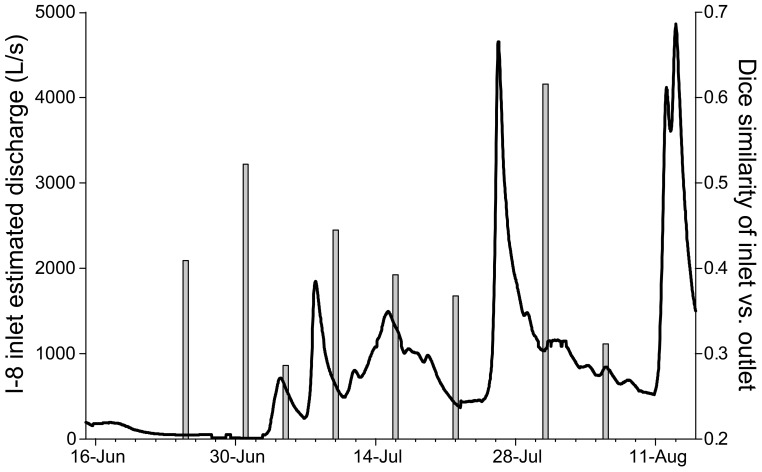
**Modeled discharge at I-8 inlet (black line) and % similarity of bacterial communities at I-8 inlet and I-8 outlet in the summer of 2003 (gray bars)**. Discharge was modeled using the relationship of discharge between Toolik Lake inlet and I-8 inlet in 2005–2007. Highest DNA similarity between I-8 inlet and outlet was detected after a large rain event near the end of July.

In order to determine the depth at which I-8 inlet water and bacteria enter the lake, a thermistor chain was deployed at the deep sampling site of the western basin (Lake I-8 W, Supplemental Figure 2). The summer of 2003 was relatively cold and wet with several large storm events (Figure 5; Adams et al., 2010). During the summer, the lake had periods of stratification until the last week of July, and had isothermal conditions for most of August and for brief periods earlier in the summer. The depth of inflow (dark columns on Supplemental Figure 2) varied throughout the summer, with base flow and early season run-off events intruding at the base of the epilimnion. Inflowing water occasionally entered a well-mixed water column or formed surface overflows. Deep intrusions to the very bottom of the hypolimnion were typically found in August when inlet water temperatures were colder (e.g., 6 August).

The spatial extent of inflowing water also affects bacterial dispersal into the lake, and the degree of mixing between inflow and lake impacts the strength of mass effects on community composition. Conductivity and pH profiles from the western sampling stations of the lake indicate that after the first small rain event of the season in 2007, the inflow signal intruded at the base of the epilimnion around 3 m, but was only detectable at the southwest station closest to I-8 inlet (Supplemental Figure 3). Presumably, this stream water mixed into the epilimnion and lost its distinct conductivity and pH signature before reaching the next sampling station.

**Figure 6:**
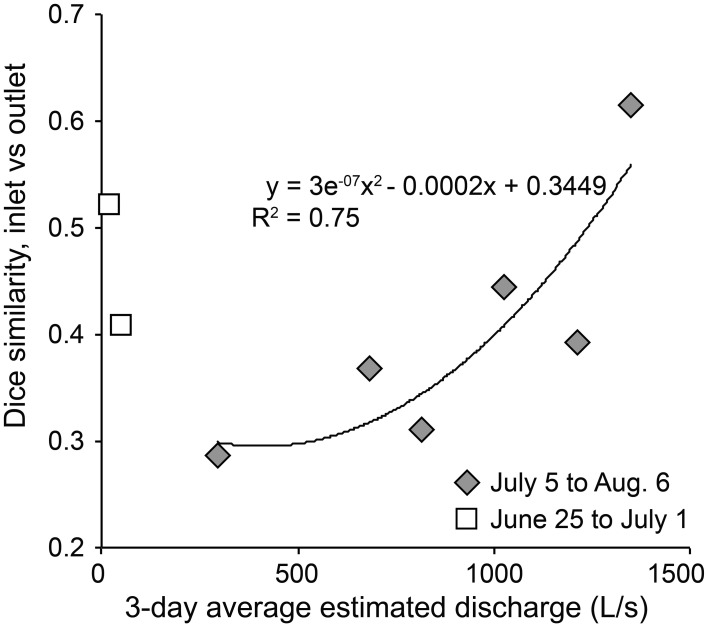
**Dice similarity between inlet and outlet bacterial community composition against the average estimated discharge for 3 days prior to community composition comparisons**. A polynomial line is fit to samples collected after July 1.

The amount of time that inflowing water persists in a habitat directly affects community dynamics by setting the amount of time available for bacterial populations to grow and overcome dilution and dispersal. Epilimnion water retention time averaged ~9 days but varied from <1 to 600 days, depending on stream discharge (Table 2). Previous estimates of WRT at this site were based on the lake-area relationship between Lake I-8 and Toolik Lake (Kling et al., 2000; Crump et al., 2007) instead of the direct measurements of discharge presented here. Bacteria cell doubling times were highly variable (Table 2). The shortest doubling time (0.4 days) was shorter than the shortest WRT (0.8 days), but the wide range of doubling times indicates that WRT was occasionally shorter than doubling time, creating a condition in which species sorting has little effect. The balance between doubling time and WRT affects the persistence of populations within the habitat. A large but variable fraction of populations in the outlet community were concurrently detected in the inlet community, ranging from 32 to 63% (mean of 48%) in 2003 and from 50 to 71% (mean of 59%) in 2007. The degree of overlap between the inlet and outlet community was related to the amount of stream flow, and thus the WRT of the lake (Figure 6), in July and August. Overall community similarity between the inlet and outlet calculated for each sampling date ranged from 29 to 62% (mean 42%) and was greatest after the largest storm event of 2003 (Figures 3, 5).

**Figure 7:**
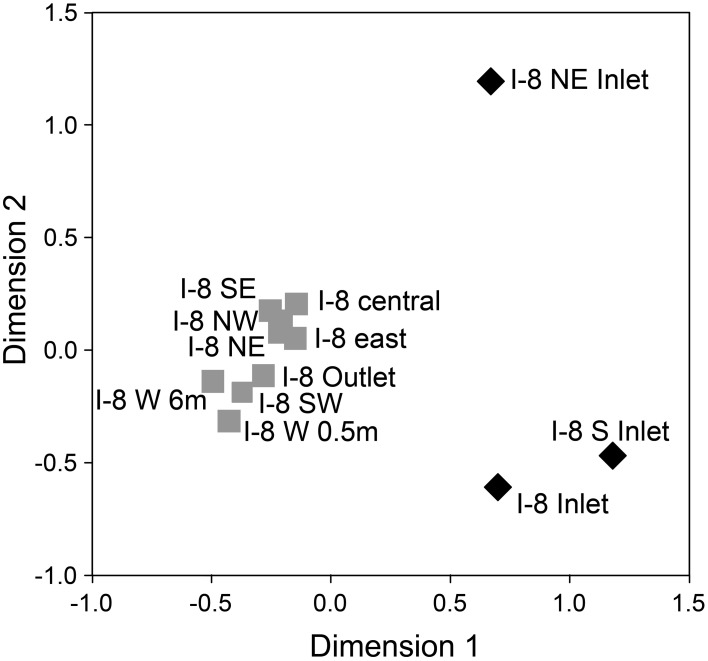
**NMDS of bacterial communities at Lake I-8 on 4 July 2007 determined from Dice similarities of 16S rRNA DGGE banding patterns for Inlet samples (black diamonds) and outlet and lake samples (gray squares)**. All samples were collected from surface water (0.01 m for streams, 0.5 m for lake) unless otherwise noted. Normalized raw stress = 0.009.

Bacterial communities collected from three inlet streams, the outlet stream, and several locations in the lake on 4 July 2007 clustered based on lotic or lentic habitat (Figure 7). Lake communities collected at several locations and depths within the lake, including I-8 outlet, had a high mean similarity to each other of 76% (± 9% *SD*), while inlet communities had a lower mean similarity to each other of 32% (± 30% *SD*) and had only 28% average similarity to the lake communities (*SD* of 9%). The two inlet streams draining catchments south of the lake (I-8 inlet and I-8 S inlet) had two-thirds of their bacterial community members in common, while the I-8 NE inlet had very low similarity to the other two inlet sites (13 and 17%, respectively).

## Discussion

Bacterial populations that are best adapted to local environmental conditions should dominate in habitats with those conditions provided that species sorting is the primary mechanism structuring the community. Thus, when dispersal is relatively low and species sorting dominates, community composition in habitats with different environmental conditions should be dissimilar. However, if dispersal is high and different habitats are linked hydrologically, then mass effects can produce greater similarity in community composition between habitats. Based on our hydrologic assessment, the inlets and outlet of lake I-8 are not highly linked except during large storm events when the communities in these two environments became more similar, demonstrating the impact of mass effects on patterns in microbial diversity over time scales of days.

The main outlet of Lake I-8 supported both greater bacterial productivity and a more stable community composition than the main inlet to the lake (Figures 2, 3). This elevated BP is likely supported by relatively labile DOM sources from autochthonous production in the lake, as indicated by higher levels of chl *a* and proteins at the outlet than the inlet (Supplemental Table 2). Community BP is not strongly correlated with temperature in these habitats (Adams, 2010; Adams et al., 2010), and a multiple regression analysis of field data showed that temperature was only one of several parameters required to explain patterns in BP including dissolved organic carbon, chlorophyll *a*, and total dissolved nitrogen and phosphorous concentrations. This work also identified multiple temperature optima within these bacterial communities (Adams et al., 2010), suggesting that rates of bacterial production are influenced by the composition of bacterial communities. The stability of the outlet community appears to be related to the retention of water in the lake. Lakes slow the transport of bacteria through catchments, and given sufficient time allow species sorting processes to control community dynamics and to result in downstream communities that are best adapted to local environmental conditions (Van der Gucht et al., 2007). However, we found that mass effects periodically disrupt the relatively stable community composition in the lake when large storm events occur, resulting in more similar communities at the inlet and outlet (Figures 5, 6). During these periods, the communities at both locations are likely to be able to process the allochthonous DOM also being transported downstream, changing the function of the ecosystem.

When transplanted, bacteria from the inlet remain active at the outlet and have equal, if not greater, activity in this new habitat (Figure 4). However, less than half of inlet bacterial populations detected as OTUs on DGGE gels persist during transport across the lake to the outlet (Figure 3, Supplemental Table 1). This suggests that the inlet bacteria brought into the lake are being out-competed by bacteria better adapted to process lake DOM and grow under lake conditions (e.g., lower nitrate concentration, higher temperature; Supplemental Table 3) (e.g., Lindström and Langenheder, 2012). Kritzberg et al. (2005) determined that lake bacterial production is correlated with autochthonous primary production, even in habitats with high levels of allochthonous (terrestrial) DOM, suggesting that lake bacteria are better adapted to grow on autochthonous organic matter than allochthonous organic matter. When communities from the Lake I-8 outlet were transplanted to the inlet habitat, in general their productivity dropped, and in the late season they were less productive than the inlet communities incubated at the same time (Figure 4). This suggests that outlet communities no longer include (in large numbers) populations of bacteria that are able to rapidly process upstream DOM and grow under inlet conditions, possibly due to species sorting in the lake during which inlet bacteria were either outcompeted or diluted to low numbers within the new lake community. These inlet bacteria, though still present in the lake, appear to be either poor competitors or potentially limited in their dispersal to the outlet habitat during low-flow periods.

The dispersal of inlet communities into the lake is limited by the depth, spatial extent, and volume of stream inflow. Although the main inlet provides the majority of water to the lake (Table 1), this water may not extend far into the lake due to mixing or insufficient volume of inflow, as observed on 4 Jul 2007 (Supplemental Figure 3), or it may flow directly to the hypolimnion where it becomes isolated until the lake mixes deeply (Supplemental Figure 2). However, during large storm events the inflow is very high and concurrent cold air and water temperatures can result in deep mixing. Large storm events also decrease the WRT, and in other systems short WRTs have been found to increase community similarity (Lindström and Bergström, 2004). Both sufficient water volume and inflow penetration into the epilimnion would be required for mass effects to have a large impact on the outlet community, such as occurred around 30 Jul 2003 (Figure 5, Supplemental Figure 2). During this large storm event (~4500 L/s discharge), the community similarity of I-8 inlet and I-8 outlet was 62% compared to 37% similarity before the storm. However, storms of such magnitude are relatively rare; only 13% of summer storm events from 1991 to 2008 had a similar or greater magnitude (Arctic LTER data).

The inlets to the lake drain sub-catchments that differ in vegetation and surface geomorphology, both of which impact bacterial communities and therefore the types of populations that can move downstream (Judd and Kling, 2002). The fact that the I-8 NE inlet had very low similarity to the other two inlet sites (13 and 17%, respectively; Figure 7) highlights that catchment-related differences in source communities can play a role in the metacommunity dynamics in a lake. However, most populations from the smaller inlets do not appear to persist in the lake, with very low similarities between inlet and lake communities. This is likely due to the small number of inlet bacterial cells becoming diluted within the lake, as seen in other studies (Lindström and Bergström, 2004), or due to unfavorable conditions during and after dispersal (Lindström and Langenheder, 2012). Lower population sizes can result in stochastic extinctions and can decrease the ability of new populations to compete with existing lake populations, although for biofilm communities assembly appears to be robust to such stochastic processes (Besemer et al., 2012). Samples collected at several locations within the lake indicated a laterally well-mixed epilimnetic bacterial community with high similarity between communities at distant sites within the lake (Figure 7). Chemical measures of lake water support this being a well-mixed habitat (Supplemental Table 3, Supplemental Figure 2) and there was no evidence for a gradient of community composition along a gradient of allochthonous inputs across the lake such as that found in a much larger reservoir by Simek et al. (2001).

In Lake I-8, there is evidence that both species sorting and mass effects structure bacterial community composition, with the impact of mass effects being limited to large stream-inflow events. Transplant experiments showed that although inlet communities have the potential for successful establishment as defined by Hanson et al. (2012) through metabolic activity in the lake and outlet habitat, outlet communities consist of many bacteria that are more limited in their ability to remain active in the inlet habitat and process allochthonous DOM (at least to the level of our detection), particularly later in the summer season. The analysis of depth, spatial extent, and volume of water inflow determined the potential dispersal of inlet bacteria to the lake. The relative magnitude of bacterial growth rates that drive species sorting, compared to physical flows and WRT which controls water-borne dispersal, determines the persistence of inlet bacteria in the lake and outlet habitat. When WRT is short and mass effects dominate, inlet stream bacteria mix farther into the lake and result in more similar community composition between inlet and outlet. WRT, along with environmental conditions, has been found to control the persistence of bacterial groups in other systems as well (Lindström et al., 2005; Crump et al., 2007), although a threshold for the importance of mass effects across habitats remains undetermined (Logue and Lindstrom, 2010). It appears that when WRT is relatively long, competition and predation structure the community in the lake, and many inlet populations do not persist or persist in very low numbers. Van der Gucht et al. (2007) suggest that high growth rates and regular dispersal allows bacterial communities to track environmental conditions but that oligotrophic systems should be more prone to mass effects than eutrophic systems with similar hydrology. In our oligotrophic study system, bacteria have a faster average doubling time than the average WRT, suggesting that species sorting dominates while mass effects may be important only during aperiodic, summer storm events. These metacommunity dynamics appear to directly influence the ecosystem function of microbial communities, by changing the species composition of communities through dispersal, and altering the ability of microbial communities to process different DOM substrates and their rates of activity under different conditions.

### Conflict of interest statement

The authors declare that the research was conducted in the absence of any commercial or financial relationships that could be construed as a potential conflict of interest.
