# Supplementary material for: Metacommunity dynamics of bacteria in an arctic lake: the impact of species sorting and mass effects on bacterial production and biogeography
Source: Front Microbiol. 2014 Mar 4;5:82. doi: 10.3389/fmicb.2014.00082 (PMC3940886; doi:10.3389/fmicb.2014.00082)
Supplement: Supplementary file 1 [file Presentation1.PDF]

Metacommunity dynamics of bacteria in an arctic lake: the impact of species sorting and mass effects on bacterial production and biogeography

Heather E. Adams[1], Byron C. Crump[2]*, and George W. Kling[1]

[1]University of Michigan, Department of Ecology and Evolutionary Biology, Ann Arbor, MI, USA
[2]Oregon State University, College of Earth, Ocean and Atmospheric Science, Corvallis, OR, USA

Supplemental Table 1. DNA similarity (1.0 = identical) between sites during transplant experiments and the impact of location on community composition (average similarity of control compared to location treatment). Samples with slashes in the name have the community source environment before the slash and the incubation environment after the slash.

Date	Samples compared		Similarity
18 July 2006	I-8 inlet	I-8 outlet	0.38
18–21 July 2006	Inlet/inlet	Inlet/outlet	0.77
21 July 2006	I-8 inlet	I-8 outlet	0.56
1 Aug. 2006	I-8 inlet	I-8 outlet	0.51
1-3 Aug. 2006	Inlet/inlet	Inlet/outlet	0.74
1-3 Aug. 2006	Outlet/outlet	Outlet/inlet	0.90
3 Aug. 2006	I-8 inlet	I-8 outlet	0.55

Supplemental Table 2. Habitat characteristics during transplant experiments. Transplant experiment collected on 9 Jul 2005 was deployed 12 hours after conditions were measured on 5 Jul 2005.

Date	**Site**	**Temp** (°C)	**BP** (μg C/L/d)	**Cond** (μS/cm)	**pH**	**Chl *a*** (μg/L)	**Protein** (mg/L)	**Phenolics** (μM)	**DOC** (μM)
5 Jul 2005	I-8 inlet	7	1.25	123.3	7.28	0.21	0.16	0.06	268
5 Jul 2005	I-8 outlet	12.3	7.19	51.2	6.99	0.39	0.29	0.04	387
9 Jul 2005	I-8 inlet	7.2	1.69	62.0	6.29	0.18	0.51	0.81	615
9 Jul 2005	I-8 outlet	10.6	11.4	62.2	6.61	0.80	0.53	0.99	457
26 Jul 2005	I-8 inlet	11.2	1.42	117.2	7.12	0.10	0.16	0.29	354
26 Jul 2005	I-8 outlet	14.6	6.38	67.9	7.61	0.75	0.38	0.78	527
28 Jul 2005	I-8 inlet	11	1.79	124.3	7.66	0.90	0.16	0.27	351
28 Jul 2005	I-8 outlet	14.4	10.6	69.5	7.54	0.97	0.34	0.76	522
18 Jul 2006	I-8 inlet	10.1	0.85	88.5	8.14	0.41	0.30	0.56	463
18 Jul 2006	I-8 outlet	12.4	2.39	69.6	7.65	2.10	0.59	1.05	574
21 Jul 2006	I-8 inlet	8.3	1.16	77.7	7.59	0.43	0.47	0.73	528
21 Jul 2006	I-8 outlet	11.6	7.70	72.5	7.39	1.72	0.59	0.95	564
1 Aug 2006	I-8 inlet	10.1	0.56	95.9	7.62	10.1	0.45	0.43	476
1 Aug 2006	I-8 outlet	12.6	3.86	75.4	7.41	12.6	0.59	0.87	592
3 Aug 2006	I-8 inlet	12.1	2.09	100.4	7.72	0.65	0.47	0.48	457
3 Aug 2006	I-8 outlet	12.8	10.8	78.4	7.52	2.10	0.62	0.81	576
2 Jul 2007	I-8 inlet	17.6	7.15	152.2	7.65	0.29	0.12	0.68	402
2 Jul 2007	I-8 outlet	16.3	15.4	79	7.47	0.48	0.19	0.69	315
4 Jul 2007	I-8 inlet	13.9	2.69	158	7.50	0.20	0.21	0.23	363
4 Jul 2007	I-8 outlet	18.4	2.68	74.2	7.25	0.29	0.32	0.03	427

Supplemental Table 3. Limnological variables measured at Lake I-8 during the 4 Jul 2007 intensive spatial sampling (see Kling et al. 2000 for methodology).

Site	Time	Depth (m)	Temp °C	Cond µS/cm	pH	Alk µeqL	DOC µM	NH_4 µM	PO_4 µM	NO_3 µM	TDN µM	TDP µM	Ca µM	Mg µM	Na µM	K µM	Si µM	Oxygen mg/L	Chl*a* µg/L
I8 Inlet	9:55	0.01	13.9	158	7.5	1418	363.3	0.35	0.02	13.82	21.1	0.08	668	146	22.3	5.4	30.5	.	0.23
I8 Inlet Northeast	9:15	0.01	11.3	208	7.53	1898	427.3	0.57	0.06	18.70	24.7	0.06	917	176	30.8	5.7	41.2	.	0.36
I8 Inlet South	14:10	0.01	16.3	143.3	6.93	1379	405.9	0.54	0.07	1.86	13.5	0.11	639	114	20.4	4.4	38.0	.	0.41
I8 Lake Central	11:08	0.5	17.8	68.4	7.57	633.3	447.4	0.26	0.04	0.06	10.8	0.09	308	66.3	12.8	7.4	16.5	8.43	1.01
I8 Lake East	10:32	0.5	17.9	68.7	7.78	650.5	456.4	0.34	0.04	0.12	12.2	0.11	309	66.3	13.4	7.7	15.9	8.74	1.02
I8 Lake Northeast	10:15	0.5	18	68.8	7.85	645	444.1	0.48	0.03	0.21	12.1	0.11	312	67	13.2	7.8	15.8	8.78	0.94
I8 Lake Northwest	12:50	0.5	17.5	68.1	7.73	640.4	405.7	0.27	0.04	0.08	11.3	0.07	305	66.1	12.8	7.4	16.4	8.73	1.05
I8 Lake Southeast	10:44	0.5	18	69	7.8	634.3	429.3	0.44	0.07	0.20	12.5	0.10	311	67.5	27.8	9.2	19.1	8.80	0.65
I8 Lake Southwest	11:38	0.5	17.7	67.8	7.63	616.1	448	0.25	0.04	0.18	11.1	0.12	305	65.8	13.3	7.5	16.4	8.48	1.06
I8 Lake West	12:12	0.5	17.6	67.8	7.76	647.2	391.1	0.28	0.03	0.11	11.7	0.08	305	66.2	12.9	7.4	16.5	8.69	0.97
I8 Lake West	12:12	6	7.76	61.8	6.82	609.9	425.9	0.52	0.05	0.32	11.8	0.14	290	62.6	13.1	8.1	29.7	7.75	1.85
I8 Outlet	11:00	0.01	18.4	74.2	7.25	622.3	393.5	0.67	0.04	0.46	12.1	0.11	316	68.5	13.1	7.5	17.9	.	0.34

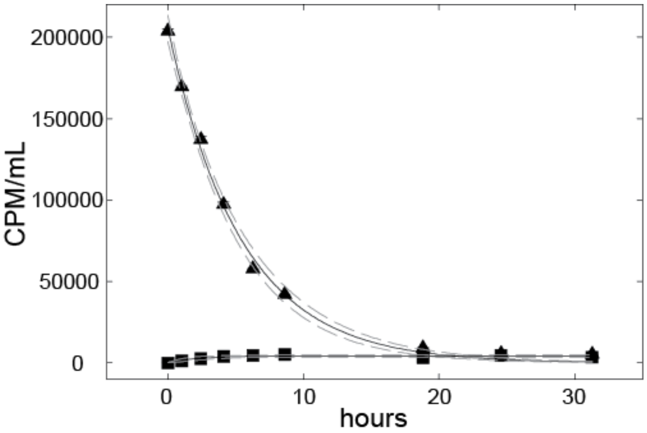

Supplemental Figure 1. Diffusion of ^{14}C-leucine across dialysis bags. Upper line (triangles) is radioactivity of ^{14}C-labeled leucine measured inside a dialysis bag (in scintillation counts per minute (CPM) per ml), while the lower line is radioactivity measured in the fluid exterior of the bag (squares). Black solid lines are fitted to the curves with exponential equations (decay and increase, R^2 = 0.997 and 0.84), and gray dashed lines are 95% confidence intervals. Error bars on data points are standard error of the mean.

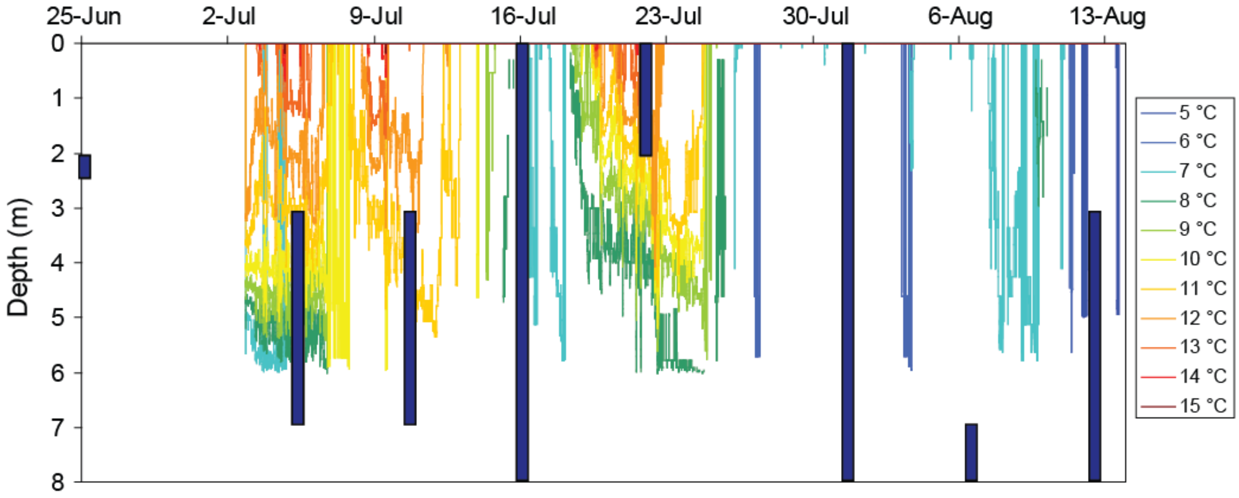

Supplemental Figure 2. Plot of isotherms in Lake I-8 from summer 2003. Bars indicate the depth range where the density of the inflow water from I-8 inlet is the same as the water within the lake (density calculated using measured temperature and salinity).

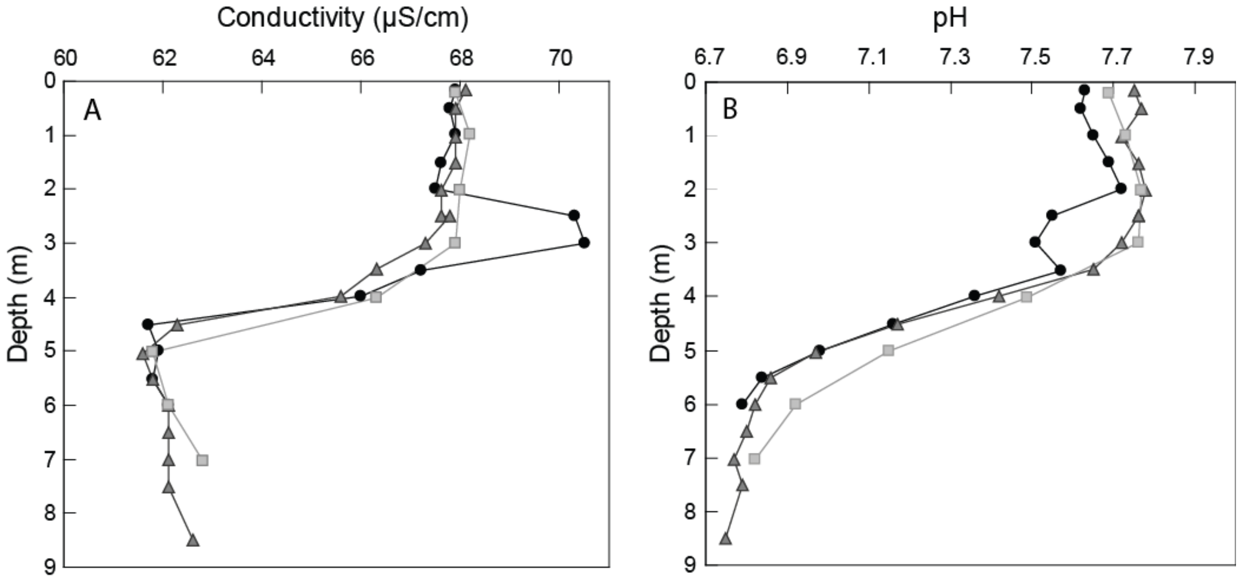

Supplemental Figure 3. Conductivity (left) and pH (right) depth profiles for the sampling transect in the western basin of Lake I-8 on 4 Jul 2007. I-8 southwest station (black circles) clearly shows the influence of the main inlet stream in higher conductivity and lower pH, compared to the west (light gray squares) and northwest (dark gray triangles) stations.
